# The impact of synchronous versus asynchronous electrical stimulation in artificial vision

**DOI:** 10.1088/1741-2552/abecf1

**Published:** 2021-04-27

**Authors:** Susana Moleirinho, Andrew J Whalen, Shelley I Fried, John S Pezaris

**Affiliations:** 1 Department of Neurosurgery, Massachusetts General Hospital, Boston, MA, United States of America; 2 Department of Neurosurgery, Harvard Medical School Boston, MA, United States of America; 3 Boston VA Healthcare System, Boston, MA, United States of America

**Keywords:** visual prostheses, microstimulation, visual perception, early vision

## Abstract

Visual prosthesis devices designed to restore sight to the blind have been under development in the laboratory for several decades. Clinical translation continues to be challenging, due in part to gaps in our understanding of critical parameters such as how phosphenes, the electrically-generated pixels of artificial vision, can be combined to form images. In this review we explore the effects that synchronous and asynchronous electrical stimulation across multiple electrodes have in evoking phosphenes. Understanding how electrical patterns influence phosphene generation to control object binding and perception of visual form is fundamental to creation of a clinically successful prosthesis.

## 1. Introduction

A promising treatment for blindness is the application of fine-grained electrical stimulation to the visual pathway that is patterned both in space a time to replace information that once originated in the eyes. Common among the varied approaches to address this challenge is the central problem of how stimulation across multiple channels is coordinated in order to evoke a predictable, coherent visual scene. Here, we present a review of the current state-of-the-art for ensuring channel independence and in particular examine the relationship of timing across simultaneously activated electrodes. In doing so, we recognize the potential impact of bipolar versus monopolar stimulation to widen the available and effective stimulation parameter space. We conclude by reviewing four open questions in the field and by proposing a behavioral experiment to examine the influences of fine-scale temporal shifts between pulse patterns applied to arrays of electrodes and thus to understand the perceptual effects of synchronous versus asynchronous stimulation.

## 2. Background

Blindness results from the inability of the visual system to transduce light into neural signals and communicate the resulting visual information to higher areas of the brain. It is estimated that 39 million are blind worldwide as a result of diseases such as cataracts, glaucoma, age-related macular degeneration (AMD), and retinitis pigmentosa (RP) (Pascolini and Mariotti 2012). While cataracts can be addressed surgically, blindness from glaucoma, AMD and RP currently have no effective surgical or medical treatment to reverse visual loss, and thus many groups are pursuing device-based therapies broadly known as *visual prostheses* to restore visual function.

Functional restoration through a visual prosthesis is possible because for many causes of blindness that render the eye insensitive to light, important aspects of the visual pathway remain intact. In the healthy visual system, the eye focuses light onto photosensitive cells in the retina that transduce photons into neural signals. After processing within the retina, the retinal ganglion cells (RGC) send the signals along their axons which exit the eye in a bundle to form the optic nerve. The optic nerve carries the visual information to the lateral geniculate nucleus (LGN) located within the thalamus. The LGN in turn sends its output through the optic radiation to the primary visual cortex, V1. From there, signals subsequently branch out to several other higher-order centers, where simultaneous, parallel analysis results in activity that embodies visual perception.

The ability to generate *phosphenes*, visual percepts that are not caused by the normal activity of the eye in response to light, has a long history of study. Mechanical stimulation has been recognized as a means to create phosphenes for thousands of years as initially described by Alcmaeon in the 5th century BC (Grüsser and Hagner 1990). Electrical stimulation, the focus of this report, has been recognized for over 200 years as initially described in 1755 by Le Roy (1755) and Walsh and Pascual-Leone (2005). Le Roy, in an unsuccessful attempt to cure blindness, applied electric shocks to a blind patient’s head, inducing temporary, vivid phosphenes. A comparative flurry of activity followed 150 years later when Foerster (1929), Krause and Schum (1933), and Button and Putnam (1962) began to explore the idea of a visual prosthesis in earnest, as described in historical reviews by Donaldson and Brindley (2016) and Lewis and Rosenfeld (2016).

It was not until the late 1960s that the first visual prosthesis was developed by Brindley and Lewin using focused electric stimulation of the brain in blind subjects (Brindley and Lewin 1968a, 1968b). Their elegant work investigated phosphenes generated through an 80 electrode grid implanted on the medial surface of the occipital pole of the right hemisphere (placed subdurally, without penetration of the cortex) in a 52 year old woman who had lost her vision as a result of bilateral glaucoma. Of the 80 electrodes, 32 could evoke individual visual phosphenes and strikingly, simultaneous stimulation of adjacent electrodes was described as evoking phosphenes that could be combined to form simple patterns such as letters. Further studies in subsequent years (Donaldson 1973, Dobelle and Mladejovsky 1974, Pollen 1975, Dobelle et al 1976) confirmed that although spatially patterned electrical stimulation of the visual cortex evoked spatially patterned percepts, predictability of phosphenes was somewhat challenging given these early electrode designs and high current levels they required.

Since then, several groups have applied their research efforts towards the development of visual prostheses that target various structures along the early visual pathway (Pezaris and Eskandar 2009). Two main structures have received particular attention: the intact inner retina (reviewed in Zrenner 2002, 2013), and the primary visual cortex (Schmidt et al 1996, Bradley et al 2005, Schiller et al 2011, Fernandez and Normann 2017). Other efforts have focused on two additional targets in the early visual pathway: the optic nerve (Veraart et al 1998, 2003, Nishida et al 2015, Gaillet et al 2020) and the LGN of the thalamus (Pezaris and Reid 2007, Panetsos et al 2011). The specific advantages and disadvantages of each location have been comprehensively reviewed elsewhere (Pezaris and Eskandar 2009, Lewis and Rosenfeld 2016, Najarpour et al 2018, Mirochnik and Pezaris 2019).

In spite of significant effort towards the development of visual prosthetics, the only systems currently approved for clinical use target the retina: Argus II, Alpha AMS, and IRIS II. The Argus II device (Second Sight Medical Products, CA, USA) uses a 60 electrode stimulating array, and has regulatory approval in both Europe (CE Mark) and US (FDA approval) (Perez et al 2012, Bloch and da Cruz 2020). The Alpha-AMS device (Retina Implant AG, Germany), uses a 1600-site microphotodiode array, and has received a CE Mark (Stingl et al 2013, Zrenner 2013). Regrettably, both of those companies have been negatively impacted by low adoption rates, in part because of limitations in the quality of restored function provided by their devices (Zrenner et al 2019). The IRIS II device (Pixium Vision, SA, Paris, France) uses a 150-microelectrode array, and has also received a CE Mark (Eckmiller et al 2005, Hornig et al 2017). Approaching regulatory approval is the PRIMA device (also by Pixium Vision) which uses a 100 *μ*m pitch microphotodiode array driven by an external infra-red projector, and is currently in clinical trials (NCT03333954, NCT03392324). In addition to these advanced retinal projects and others (e.g. Degenaar et al 2009, Shivdasani et al 2010), several clinical trials are currently ongoing for cortical devices (RNS System, NCT02747589; CORTIVIS, NCT02983370; Orion, NCT03344848; see review in Mirochnik and Pezaris 2019). Despite active pre-clinical efforts in optic nerve (Nishida et al 2015, Gaillet et al 2020) and LGN devices (Kyada et al 2017), the preferred target in the field continues to be the retina (reviewed in Bloch et al 2019, Mirochnik and Pezaris 2019), although recent advances in cortical stimulation may indicate a shift toward cortical devices (Chen et al 2020). For the purposes of this review, we will be taking an agnostic position on device design to discuss stimulation issues that should apply to any approach.

### 2.1. Controlling individual phosphenes

It is assumed when designing and engineering the features of visual prostheses that any given electrode evokes a phosphene characterized by a relatively consistent region of the visual field determined by the retinotopic map of the implanted area, as has been verified in animal models (Bradley et al 2005, Pezaris and Reid 2007, Chen et al 2020). Modulation of stimulus parameters have been found to affect phosphene characteristics, allowing modest control over size, brightness, duration, or color (e.g. Brindley and Lewin 1968b, Dobelle and Mladejovsky 1974, Schmidt and Bak et al 1996, Veraart et al 2003, Greenwald et al 2009, Schiller et al 2011, Nanduri et al 2012, Winawer and Parvizi 2016). The extent of control differs depending not only on the parameters applied during the microstimulation, but also on the configuration and location of the implanted electrodes (Torab et al 2011, Najarpour et al 2018). Electrically-evoked individual phosphenes are generally found to be small, mostly round or punctate, fuzzy-edged, white-to-yellowish in color, and static, but it is important to recognize that there are exceptions reported to each of those broad characteristics. Phosphenes are generally created by stimulation in the range of a few tens of microamperes of current for penetrating electrodes (e.g. Schmidt et al 1996, Bradley et al 2005, Schiller et al 2011, Chen et al 2020), to an intermediate range for small contact size surface electrodes (e.g. Veraart et al 1998, Wilke et al 2011, Lorach et al 2015, Christie et al 2016), to a few milliamperes for large surface electrodes (e.g. Dobelle and Mladejovsky 1974, Beauchamp et al 2020), in a short train of charge-balanced biphasic pulses (ones with equal total current flowing in positive-going and negative-going phases). With these predictable characteristics, phosphenes can be considered to be the pixels of artificial vision, although it is also important to understand that for many contemporary visual prostheses, phosphenes are typically spread apart in the visual field rather than close-packed in a contiguous plane (see Pezaris and Reid 2009 for example distributions).

Phosphene threshold, the minimum stimulation intensity capable of reliably eliciting a phosphene, is the fundamental parameter for generating phosphenes. It is influenced by several mechanical factors such as electrode size and proximity to tissue (Kasi et al 2011, Christie et al 2016), generally following the path that larger or more removed electrodes require higher stimulation levels (e.g. Brindley and Lewin 1968a, Dobelle and Mladejovsky 1974, Humayun et al 2003, Shivdasani et al 2014, Beauchamp et al 2020) than smaller and more intimately placed ones that require lower stimulation levels (e.g. Schmidt et al 1996, DeYoe et al 2005, Wilke et al 2011). It is also influenced by several electrical factors such as pulse amplitude, duration, count and repetition rate that interact through strength-span curves that generally allow trading one parameter off for another (e.g. Brindley and Lewin 1968b, Dobelle and Mladejovsky 1974, Schmidt et al 1996, Delbeke et al 2003, Rizzo et al 2003, Horsager et al 2009, Klauke et al 2011, Nanduri et al 2012, Shivdasani et al 2012, Lee et al 2013, Najarpour et al 2018). Limits to avoid long-term tissue damage at the interface set an upper bound on the level of current that can be safely delivered (Cogan 2008), but fortunately, with clever pulse design it remains possible to reliably elicit phosphenes without violating these limits (Troyk 2011).

### 2.2. Controlling multiple phosphenes and creating the perception of visual form

Given the ability to create individual phosphenes, understanding how to combine individual phosphenes into an image becomes pivotal to the goal of restoring visual function. The idea that a large number of phosphenes can be combined to create a device with high utility has support from simulation studies (Bourkiza, Vurro et al 2013, Vurro et al 2014, Rassia and Pezaris 2018), and resonates with our intuitive understanding of visual function, but requires that these phosphenes be under independent control, and, importantly, be coordinated through patterned stimulation to create perceptions of objects, or *form vision*.

**Figure 1.**
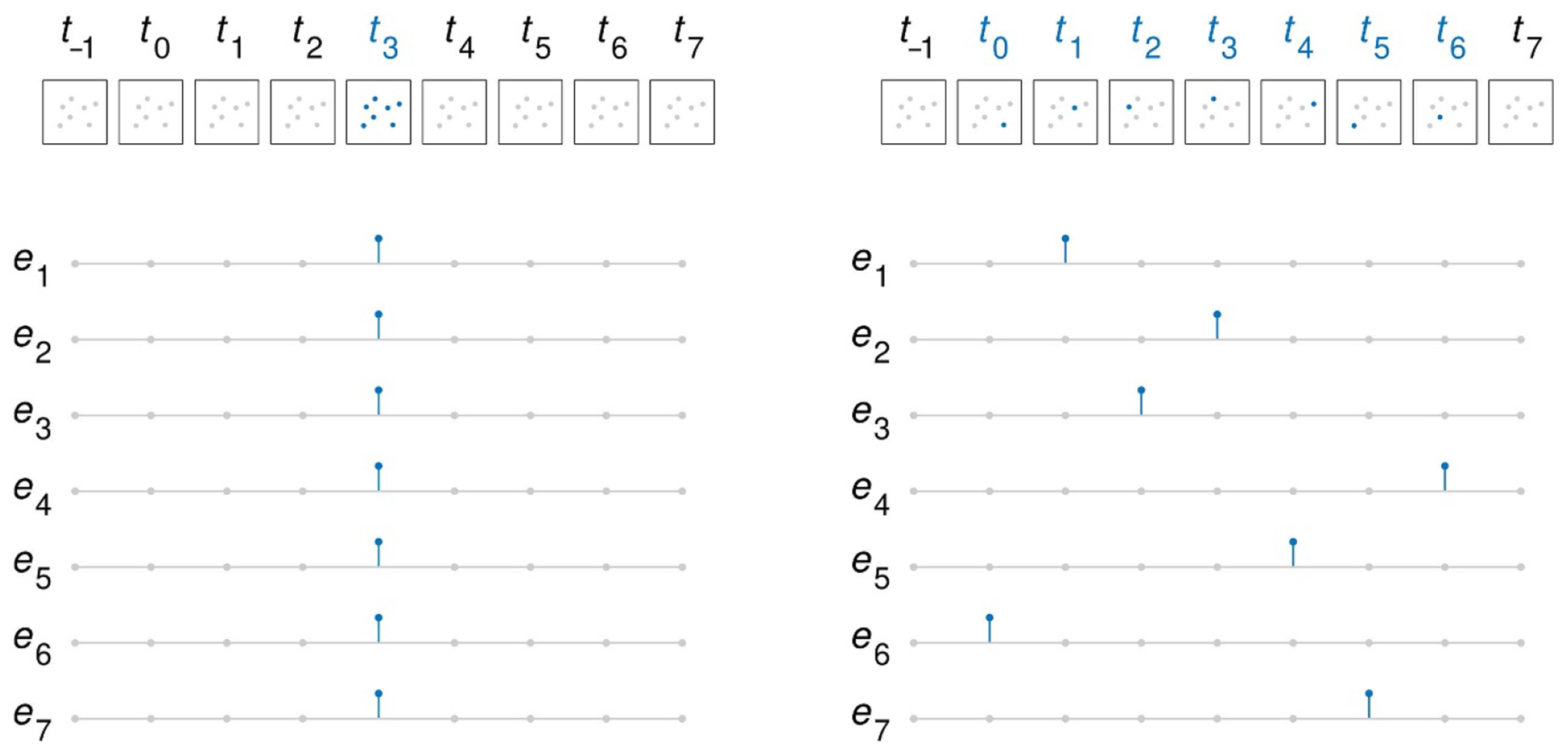
Synchronous and asynchronous stimulation of a square figure. The time courses of seven hypothetical phosphenes and the electrode stimulation used to create them are shown in the upper and lower parts of the figure, respectively. Each frame across the top of both left and right subfigures represents a single time period, perhaps a millisecond long or less. Each dot represents the visual field location of a phosphene, gray for unstimulated, blue for stimulated. Each trace across the bottom corresponds to the stimulation pulse applied to an electrode over time, gray for disabled, blue for enabled. Merely the presence or absence of pulses are depicted, rather than the exact, presumably charge-balanced pulse shapes. While additional, unactivated electrodes and their phosphenes are not shown, this cluster of seven can be used to convey a roughly square figure. (**left**) Synchronous stimulation is applied to all seven electrodes at time *t*_0_, triggering the phosphenes for all seven simultaneously. Potential unintended phosphene-phosphene interactions are not shown. (**right**) Asynchronous stimulation is multiplexed across the seven electrodes from time *t*_0_ *t*_6_ to reduce or eliminate unintended phosphene–phosphene interactions. Whereas synchronous stimulation can be applied with an instantaneous latency, asynchronous stimulation requires a temporal spreading of the stimulation. Rigorous investigation of the relative effects of synchronous versus asynchronous stimulation would need to examine not just the efficacy of evoking the desired shape (through for example, percentage correct through a two-alternative forced-choice paradigm), but also the latency of recognition (as reaction time), to find an appropriate balance between reduction of unintended phosphene-phosphene interactions and enhancement of representational phosphene-phosphene synchronicity to induce object binding.

In particular, we need to understand the effects of synchronized (simultaneous) versus unsynchronized (sequential) stimulation of multiple microelectrodes on the perceptual binding of the separate phosphenes they generate into a cohesive object. When applying multi-electrode stimulation, although stimulation parameters (pulse count, waveshape, amplitude, duration, inter-pulse interval) might be kept consistent across different electrodes, the fine-scale temporal relationship of individual pulses across electrodes has been shown to result in differing perceptual effects, as will be discussed below. These patterns consist of either synchronized stimulation with pulses that are simultaneously presented within fewer than perhaps tens of microseconds of each other, or asynchronous stimulation with pulses in a phase-shifted pattern such that they do not temporally overlap (Cicione et al 2014, Hokanson et al 2018), as shown in figure 1.

Some reports of successful form vision have employed synchronous stimulation. For example, the Alpha AMS, a subretinal micro-electrode array with 1600 active microphotodiodes, has restored visual perception of single letters and Landolt C rings in patients blind from hereditary retinal degeneration (Zrenner et al 2011). Similarly, an experimental mode of the Argus II, also in a patient blinded by outer retinal dystrophy, used synchronous stimulation to allow the identification of 89% of single letters, 60% of three-letter, and 70% of four-letter words (Lauritzen et al 2012), however production versions of Argus II are believed to use asynchronous stimulation. Most recently, the PRIMA device, a subretinal array triggered by pulsed illumination that creates synchronous stimulation of the retina (Lorach and Palanker 2016), was implanted in three patients blinded from AMD and, with 378 microphotodiodes at 100 *μ*m separation, supported letter identification with Landolt C acuity of logMAR 1.45 ± 0.10 (Palanker et al 2020), which translates to 20/560 mean and 20/450–20/710 standard range on the Snellen scale.

### 2.3. Field interactions during synchronous and asynchronous electrical stimulation

To examine the link between synchronous stimulation and its perceptual impact, we start with existing work in other fields. The impact of electric field interactions between electrodes during simultaneous stimulation is considered a central problem in other sensory prostheses such as cochlear implants (Cicione et al 2014, Hokanson et al 2018). Although cochlear implants for restoring hearing to the deaf do not typically penetrate the neural tissue of the cochlea (Zeng et al 2008), they provide an excellent example of the pitfalls that must be considered when designing visual prostheses. As electric fields in a linear medium obey the *principle of superposition*, whereby the total field from a suite of electrodes is the simple sum of the field from each individual electrode (e.g. Seibert 1986), we might assume that the effects of simultaneous stimulation on cochlear neuron activation similarly would be the simple sum of neural activity from stimulation of individual electrodes. But that does not appear to be the case due to the non-linear response characteristics of cochlear neurons whereby combined electrical stimuli counter-intuitively produce detrimental perceptual changes due to strong channel interactions (Shannon 1983, De Balthasar et al 2003) limiting the number of independent channels to 7–10 even for 24-channel implants (Fishman et al 1997, Friesen et al 2001). Helping to explain this observation, the neural response as assessed by the number of cells recruited at the active sites of cochlear electrodes was found to be non-linear and significantly higher than with asynchronous stimulation (Hokanson et al 2018). The idea that the principle of superposition does not apply to the neural response to electrostimulation is found in other structures as well (Histed et al 2009, Jepson et al 2014a). An effective approach to address this interaction problem for cochlear implants is the delivery of interleaved, non-simultaneous pulses to each electrode to eliminate temporal overlap across channels, which improves the predictability of the evoked auditory percept and results in higher device utility (Shannon 1983).

Asynchronous pulse-delivery has been independently investigated for peripheral nerve applications and reported as beneficial in both tissue-penetrating microelectrodes (Hokanson et al 2018) and non-penetrating electrodes (Strauss et al 2019). Advantages were seen for electrode separations at microscopic (400 *μ*m in a Utah array: Normann et al 2012) and macroscopic scales (separate nerve bundles: Hokanson et al 2018, Strauss et al 2019). Disadvantages found, however, were that significantly higher asynchronous stimulation was required to achieve the same functional or perceptual result as synchronous stimulation. An informative exception to that finding is when using functional electrical stimulation (FES) to control muscular output: interleaved stimulation produces much more fatigue-resistant force than synchronous stimulation. FES, unlike sensory stimulation, exploits the many-to-one neuromuscular architecture where multiple nerve fibers act in parallel to control the contraction of a single muscle.

Returning to visual prostheses, practical issues may limit the applicability of sequential stimulation. With sequential stimulation, each electrode is typically assigned a time slice, or multiplexing period, when stimulation is to be applied within the frame cycle. Longer multiplexing periods imply slower frame rates, which can lead to a poor patient experience due to factors such as fading (Perez et al 2012) or blinking of percepts (Zrenner et al 2011). Thus the visual prosthesis designer generally seeks to implement as fast a frame rate as possible, ideally above flicker fusion which can range 10–60 Hz (Schmidt et al 1996, Veraart et al 2003, Horsager et al 2010, Goetz et al 2015, Lorach et al 2015), or perhaps higher (Brindley and Lewin 1968a). With fast frame rates, the restricted sequential time slice available for each electrode to evoke a phosphene is complicated by compliance-voltage limits that disallow extremely short, high-amplitude pulses when delivering a given charge, out of concern for long-term damage to tissue and electrodes (Shannon 1983, Merrill et al 2005). With the two competing constraints of very short multiplexing periods for each electrode in high-density implants pressing towards brief, tall pulses and so-called water-window (Cogan 2008) limitations pressing towards longer, weaker pulses, there appears to be an impasse for sequential stimulation. For low-resolution devices with low refresh rates, such as Argus II or Orion, there is about a 2 ms window per electrode (60 electrodes with 8 Hz refresh rate); but for a high fidelity device with 1000 electrodes and 30 Hz refresh, the window is 33 *μ*s. To deliver the same amount of charge in the high-resolution case would require much stronger current levels, which in turn can create undesirable effects such as a suppressive halo around phosphenes (Humayun et al 2003, Barriga-Rivera et al 2017), complete scotoma (Logothetis et al 2010), or even tonic silencing of visual cortex (Kara et al 2002), in addition to potentially triggering tissue or electrode damage.

Another issue that may potentially vex asynchronous stimulation is that of apparent motion. Two nearby phosphenes presented simultaneously may appear as two individual percepts, or may fuse together to a larger whole (Brindley and Lewin 1968b). In contrast, the same two phosphenes when stimulated in rapid succession might be expected instead to trigger a sense of apparent motion (Julesz and Payne 1968, Anstits 1970), and in some cases has been done so intentionally (Humayun et al 2003, Shivdasani et al 2017, Titchener et al 2020, Beauchamp et al 2020). If that sequence applies to the full extent of the prosthesis visual field as a rasterized scan, the apparent motion may become overwhelming unless the scan is either broken up into regions, or performed quickly enough to avoid motion artifacts. We speculate that reports have not appeared on this latent issue as contemporary visual prostheses provide insufficiently high resolution to reveal such effects, but as long as the refresh rate on a scanned array is comparable in speed to the motion of objects in the environment or the subject through the environment, it remains a potential pitfall.

**Figure 2.**
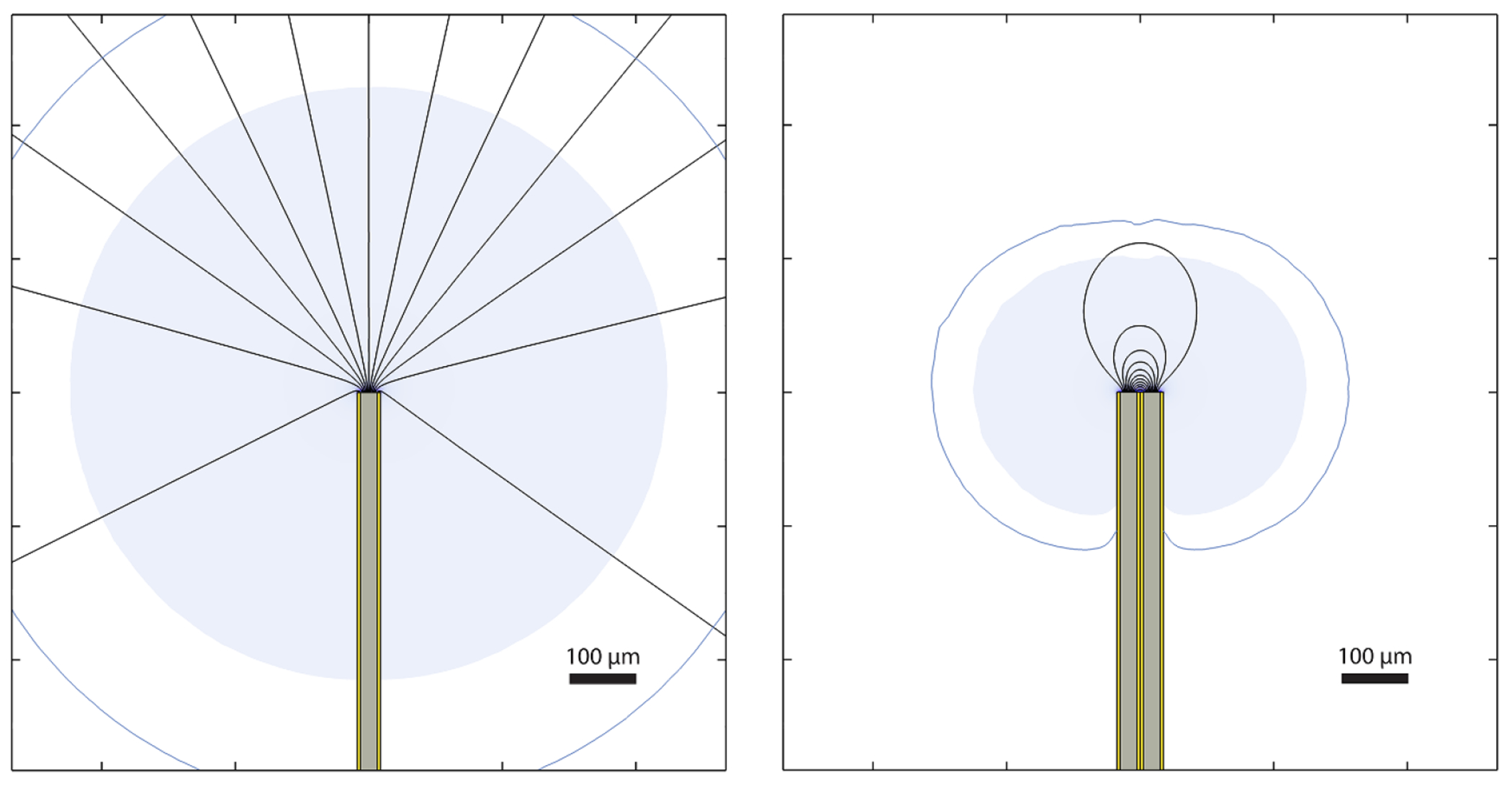
Monopolar versus bipolar electrode configurations. The electric fields that stimulate neurons in a visual prosthesis are highly dependent on their configuration. In this simulation, we have placed electrodes in a uniform, homogeneous material; while there are substantial differences with examples where electrodes are placed on sheet-like tissue (such as the retina or cortical surface), the fundamental observations are the same. Electric fields and the currents they generate are always applied between two conductors, although in many neurostimulators, a large, distant common return contact is shared across electrodes in what is often called a *monopolar* configuration with remote return (left). In such applications, the fields diverge from the electrode tip and fall off with distance *r* proportional to 1/*r*^2^. Only the volume of tissue around the electrode tip has sufficient current density to be activated, but for dense sets of electrodes, these activation volumes from each tip can overlap, leading to crosstalk and unintended phosphene-phosphene interaction. A more focused field can be produced by applying stimulation between pairs of electrodes (right) at the cost of requiring twice as many wires for the same number of phosphenes. In these *bipolar* configurations, the fields are more concentrated, falling off proportional to 1/*r*^3^, and the volume of tissue with sufficient current density to be stimulated is substantially smaller, improving the independence of each phosphene. Some stimulation strategies for planar applications (like the retina or cortical surface) use hexagonal arrays of electrodes with independent control of each, allowing for a smooth transition between these two conditions, potentially allowing for a variety of phosphene sizes and locations to be generated. In the simulation depicted here, 25 *μ*m Pt/Ir wires (gray) have been insulated with 2 *μ*m HML (gold), blunt cut, placed in a medium with 3.6 Ωm conductivity (Aberra et al 2020) that simulates gray matter (white background), and stimulated with 10 *μ*A of current. Isocurrent traces (black lines) and electric field magnitudes above 10 V m^−1^ (blue shading) depict current flow and activated tissue, respectively (Chan and Nicholson 1986). Electric fields between 5 V m^−1^ (blue contour) and 10 V m^−1^ could potentially activate tissue if overlapped from a second set of electrodes.

One solution to the conundrum presented by these issues is to limit the extent of electrical fields during stimulation and thereby reduce the effects of functional cross-talk by using what is often called a *bipolar* electrode configuration that places return contacts in close proximity to driven ones, rather than using a remote, common return, often called a *monopolar* configuration, like the systems described above (see figure 2). Lovell and Suaning’s group (Dommel et al 2009) developed a retinal microstimulator featuring simultaneous stimulation of multiple electrodes with precisely localized fields featuring this technique. Their multiplexing system uses a hexagonal mosaic arrangement of the electrodes, wherein each electrode in the mosaic can become either a stimulating or return electrode to focus the stimulating field and reduce fringe field effects. This approach reduced undesirable effects of cross talk in the formation of the visual percept (Dommel et al 2009, Wilke et al 2011, Schmid et al 2013, Sinclair et al 2016). A similar approach has been studied in various forms by Palanker’s group (Lorach et al 2015, Goetz and Palanker 2016, Flores et al 2018, Ho et al 2019), and was successfully used in thalamic visual prosthesis research (Pezaris and Reid 2007). Understanding the applicability of bipolar versus monopolar electrode configurations is considered an important issue not only in visual prostheses (Eiber et al 2013), but in motor/somatosensory prostheses (e.g. Zaaimi et al 2013, Kim et al 2015), cochlear implants (Shannon 1983, Townshend and White 1987, De Balthasar et al 2003, Zeng et al 2008), and deep brain stimulation (e.g. Frysinger et al 2006).

In addition to placing the return electrode in close proximity to the stimulating contact, careful choice of the stimulation signals can help focus the effect of stimulation by controlling the part of cells or cell type that is activated (Cai et al 2013, Twyford et al 2014 Weitz et al 2015, Oesterle et al 2020; see also section 2.4 below). As mentioned earlier, adjustment of contact size will affect the extent of stimulated tissue. Although larger contacts generally stimulate larger extents of tissue than smaller ones, even with micron-scale electrodes the results can be surprising due to the combined effects of somatic, axonal and trans-synaptic activation (Histed et al 2009).

A promising solution to limiting the extent of activation is the use of magnetic stimulation from implantable micro-coils (Bonmassar et al 2012). Unlike traditional microelectrodes in unipolar configuration, the electric fields arising from micro-coils are spatially asymmetric and can therefore be used to selectively target specific neuronal subpopulations within a given region. In the cortex for example, micro-coils can activate vertically-oriented pyramidal neurons without simultaneously activating horizontally-oriented passing axons; as a result, the spatial extent of activation is much more focal (Lee et al 2016). The use of this kind of inductive activation through micro-coils may also alleviate many of the safety and stability concerns associated with micro-electrodes, although additional work is required to establish its viability.

Synchronous stimulation is implicitly used in photovoltaic visual prosthesis systems like Alpha AMS and PRIMA, but is not controllable within the array in the current form of those devices (Lorach et al 2015, Daschner et al 2018). For systems that include an external processor and stimulating electrode arrays, such as the Argus II and Orion (Bloch and da Cruz 2020), IRIS II (Hornig et al 2017), Australian bionic eye (Shivdasani et al 2010), CORTIVIS (Fernandez and Normann 2017), and so forth, the precise timing of the stimulation pattern is potentially under complete control. With that control comes the ability to precisely determine the synchrony or asynchrony of stimulation pulses across electrodes.

### 2.4. Biophysical and synaptic influences

Most visual prostheses rely on the assumption that individual electrodes activate only nearby neurons to create a small, focal phosphenes that can be assembled into images by simultaneously activating the appropriate pattern of electrodes. Unfortunately, phosphenes are not always simple, small and focal (e.g. Brindley and Lewin 1968a and 1968b, Nanduri et al 2008, 2012) nor do they reliably assemble in ways that match the pattern of stimulation (Schmidt et al 1996, Rizzo et al 2003, Klauke et al 2011, Humayun et al 2012, Stingl et al 2013, Beauchamp et al 2020), potentially resulting in muddled or low-contrast percepts (Humayun et al 1999, Yanai et al 2007).

To create precise and predictable phosphene patterns, we must face the challenge presented by the combination of the high sensitivity of neuronal axons to electric stimulation with the potentially expansive axonal extent. Electrical stimulation has been well studied in the retina, where the distal axon is found to be only slightly less sensitive than the highest-sensitivity region proximal to the soma (Jensen et al 2003, Sekirnjak et al 2008, Fried et al 2009). The axons of the RGC layer, coursing in a fan-shaped pattern across the retinal surface to become the optic nerve, have been identified as the cause for retinal implant recipients often reporting arcuate or elongated percepts that appear to follow axonal morphology (Nanduri et al 2008, Beyeler et al 2019), especially when combinations of electrodes are activated (Fine and Boynton 2015). Axonal stimulation has been implicated in causing sparse, distributed and inconsistent activity patterns in the mouse visual cortex (Histed et al 2009), raising concerns about potential performance limitations of prostheses using penetrating electrodes, although recent results from microelectrode arrays implanted in non-human primates (Chen et al 2020) and surface arrays implanted in humans (Beauchamp et al 2020) suggest that very simple shapes can be correctly identified.

Efforts to map the sensitivity of individual spiking neurons to electric stimulation versus electrode position found that activation thresholds were lowest in a dense region of voltage-gated sodium channels within the axon initial segment (AIS) (Ranck 1975, Fried et al 2009). The AIS is the site at which spikes are initiated during normal physiological function (Stuart et al 1997), and its properties determine electrical stimulation thresholds (Jeng et al 2011, Werginz et al 2020). The effectiveness of short-duration pulses of under a millisecond is consistent with the fast activation times of voltage-gated sodium channels and their sensitivity to rapid changes in extracellular electrical fields.

In the retina, non-spiking neurons are best activated by stimuli that are slightly longer at 4–10 ms (Fried et al 2006, Freeman et al 2011, Boinagrov et al 2014, Im and Fried 2015). This observation is consistent with the longer time constants of voltage-gated calcium channels in axon terminals (Freeman et al 2011) and the relative lack of voltage-gated sodium channels in non-spiking neurons, suggesting that long duration pulses work by triggering synaptic release from targeted neurons. Thus, stimulating with longer pulses largely avoids passing axons, better confining activation to a focal region around each electrode (Freeman et al 2011, Twyford and Fried 2016). These results help explain retinal implant users reporting that percepts from longer pulses are more focal than those from shorter pulses (Weitz et al 2015).

Long pulses introduce new challenges however as they have been shown to induce prolonged inhibitory signals that can adversely affect responses to subsequent stimuli, identified at the single cell (Freeman et al 2011, Jepson et al 2014b) and network (Im and Fried 2016, Im et al 2018), and ensemble levels (Logothetis et al 2010). In the retina, long-duration stimuli have been shown to elicit a sustained inhibitory input to ganglion cells (Fried et al 2006, Margalit and Thoreson 2006), almost certainly from the activation of nearby amacrine cells. In the primary visual cortex, post-stimulation suppression similarly arises from activation of nearby inhibitory neurons (Berman et al 1991). These effects likely mediate the suppressive halo around phosphenes mentioned above.

### 2.5. Network influences

The potential for reduced phosphene independence during synchronous stimulation can be understood by considering the subthreshold regions that extend beyond the threshold perimeter at each electrode (figure 2). If two electrodes are placed such that their subthreshold extents overlap, then a cell can be unintentionally activated by simultaneous stimulation of the two electrodes despite being not activated by either one alone. The actual subthreshold extents are determined in part by the morphology of cells in the region including the span of their dendritic and axonal processes. In practical scenarios the involvement of cytoarchitectural effects means that the volumes depicted in figure 2 might underestimate actual regions of influence, which could be irregularly shaped as suggested by behavioral (Beyeler et al 2019) and electrophysiological reports (Histed et al 2009). A related effect has been reported for unipolar stimulation where increased stimulation did not merely intensify the primary phosphene but engaged additional, separate phosphenes (Brindley and Lewin 1968a and 1968b) as the extent of superthreshold stimulation grows.

Network-mediated factors that influence responses to artificial stimulation continue to be identified. For example, non-linear integration in Y-type ganglion cells allows alternating stimulation from two nearby electrodes to maintain a baseline suppression that facilitates replication of many physiological signaling properties (Lorach et al 2015). Current steering through weighted simultaneous stimulation in adjacent electrodes can also be used to modulate the focus of stimulation (e.g. Jepson et al 2014a, Beauchamp et al 2020). Conversely, temporally interleaved stimuli from closely spaced electrodes may result in some axons (or cells) experiencing relatively high rates of stimulation (Cai et al 2011, Twyford et al 2014), raising the possibility of suppression responses that can be tailored to individual types (Jepson et al 2014a). But we must be mindful with retinal stimulation in particular that abnormal network integration in the degenerate retina may lead to undesirable or unpredictable results (Ho et al 2020).

### 2.6. Perceptual consequences of synchronous versus asynchronous stimulation

Several studies have focused on determining the quality of evoked phosphenes when applying simultaneous versus asynchronous stimulation using different types of visual prostheses. The first specific mention is from Brindley’s laboratory (Donaldson 1973) where their second patient was able to read Braille letters as presented through six phosphenes: the patient’s performance was 56% correct with 600 ms of synchronous stimulation, versus 79% with sequential stimulation of 100 ms per phosphene. Their report suggests that reducing phosphene-phosphene interaction through asynchronous stimulation is the primary cause of improvement, although a modern interpretation might suggest that training effects (such as seen in Rassia and Pezaris 2018) also played a role. In more contemporary work, Horsager *et al* (Horsager et al 2010, 2011) determined the perceptual impact of synchrony in two subjects implanted with epiretinal 4 × 4 electrode arrays. The most important finding of their work was that subjects were able to discern synchronous from asynchronous stimuli in a sequential A/B test, even for submillisecond phase delays in the asynchronous case. As their subjects did not report flicker for at or above 40 Hz base frequency, synchronicity must have affected some other perceptual characteristic such as brightness, shape, or presence of scintillation. Supporting the idea that brightness would have been different in the two conditions, they found in a separate, brightness-matching experiment that synchronous pulse patterns elicited much brighter visual percepts than asynchronous ones; asynchronous stimulation required nearly double the current to match the same level of brightness (Horsager et al 2010), and the amount of effective dimming increased with phase delay, suggestive of a suppressive effect (Horsager et al 2011). A similar reinforcement in synchronous cases has been reported for peripheral nerve stimulation (McDonnall et al 2004, Normann et al 2012, Strauss et al 2019).

Placing a limit on the spatial extent of electrode-electrode interactions during synchronous retinal stimulation, subjects were less and less able to distinguish between high frequency (80 Hz) synchronous and asynchronous stimulation of four points in a square as the physical distance between selected electrodes increased, falling to chance above 1.6 mm (Horsager et al 2010). In separate but similar work, Zrenner’s group reported the application of asynchronous stimulation resolved distortions to shape appearance observed during synchronous retinal stimulation (Wilke et al 2011). To achieve precise perceptions, interstimulus intervals above 100 ms were required, but importantly, very short intervals (0–100 ms) were not systematically investigated (Wilke et al 2011), making their work not directly comparable to the results above.

When considering the perceptual impacts of synchronous versus asynchronous stimulation, it is informative to consider the similar problem of global versus rolling shutters in image sensor design (Corcoran and Bigioi 2016). With global shutters, the entire visual scene is captured synchronously, whereas with rolling shutters the scene is captured asynchronously from one edge of the sensor to the other, one raster line at a time. The phase-delay effects of rolling shutters create a wobble-like fluidity of images during camera motion. We would expect to see a similar effect with asynchronous, raster scanning stimulation in a visual prosthesis for the same fundamental reason: the slight temporal decoupling of spatial information (where the camera is pointing) from visual information (what it sees) creates visual skew (Paraskevoudi and Pezaris 2019). Although brightness is the most parsimonious explanation for Horsager’s A/B results (Horsager et al 2010), wobble may have been a secondary discriminant, since subjects were instructed only to determine same or different in that test.

### 2.7. Achieving simultaneity

Synchronous stimulation with multiple electrodes can be achieved with, broadly, two distinct strategies: either parallel, independent pulse-shape control using multiple voltage/current sources (Brindley and Lewin 1968a, Caspi et al 2009, Horsager et al 2010) or ganged stimulation where a group of electrodes is connected by a single source and shares the current within the group (Wilke et al 2011). The parallel, independent stimulation approach permits precise control of the charge induced in each electrode, allowing for individual adjustment for threshold variability and safety limits across electrodes. It also supports the use of neighboring electrodes as current returns or guard rings to help limit current spread (Wilke et al 2011). On the other hand, a ganged stimulation device can be designed with substantially simpler circuitry. Ganged stimulation has been found to induce lower perceptual thresholds compared to stimulation of single contacts in patients with suprachoroidal retinal prostheses (Shivdasani et al 2014), consistent with synchronous stimulation in general.

Even with synchronous stimulation, there will be temporal interactions for a given electrode from one pulse to the next, especially if a prosthesis uses frame-by-frame updating of stimulation. The timing between individual electrical pulses has been shown to affect RGC activity in retinal prostheses (Jensen and Rizzo 2007, Weitz et al 2015). For example, the presentation of two pulses of equal strength separated by less than 25 ms resulted in a reduced probability of evoking an action potential by the second pulse compared with the first (Jensen and Rizzo 2007). Applied across multiple, neighboring electrodes, this efficacy-reduction effect has been used to explain perceptually detectable changes in stimuli for near-synchronous stimulation when reversing pulse sequence order (Horsager et al 2010). The same effect could partially explain reports from blind patients on the fading of phosphenes upon continual electrical stimulation (Bartlett et al 2005, Brindley and Lewin 1968a, DeYoe et al 2005, Dobelle and Mladejovsky 1974, Rushton and Brindley 1978).

### 2.8. Synchrony implicated in object perception

One of the most compelling proposed neural signaling schemes is rank-order encoding (Thorpe and Gautrais 1998; reviewed in Van Rullen and Thorpe 2002), founded on the premise that neurons in the early visual pathway that receive their optimal input will be first to spike with the presentation of a new scene (in intact organisms, rather than through visual prostheses). For example, edge detectors that have an edge fall exactly in place with exactly the right orientation will fire faster than those that are ever-so-slightly mismatched; such optimally stimulated cells will fire together to form, for example, the outline of an animal (Van Rullen and Thorpe 2001). The first cells to fire will be those delivering the most well-matched messages about image characteristics, making the precise order of spike arrival of critical importance (e.g. Guetig et al 2013, Butts et al 2007). This theoretical scaffolding has found extensive experimental support through both physiological (Thorpe et al 1996, Liu et al 2009) and behavioral (Kirchner and Thorpe 2006, Crouzet et al 2010) measurements of visual discrimination that imply the feed-forward signal path must be supporting high-level decisions with only enough time for the first spikes to arrive. An important implication of this work is that information within the visual system may best be expressed in the time domain (Branco et al 2010, Guetig et al 2013), and, for the present report, that synchronously evoked spikes, like the wave from first-firing cells across the visual field, carry more weight than asynchronous ones in object perception (Singer 1999).

### 2.9. Open questions on synchronicity in artificial and normal vision

As electrode count increases with advances in visual prostheses, the need to address the issue of synchronicity in stimulation becomes more pressing as the field makes strides toward high-fidelity artificial vision. As described above, the primary impediment to fully independent control of electrodes that would support application of stimulation at arbitrary times free from concerns of undesirable phosphene effects is the potential for overlap among the regions of tissue influenced by each electrode. Designs that limit the extent of tissue that is just below stimulation threshold are advantageous in that regard (figure 1; Palanker et al 2005, Pezaris and Reid 2007, Matteucci et al 2016). But, given the freedom to stimulate or not stimulate on a fine time scale, what should designers chose? In the paragraphs below, we list four open questions and, in the next subsection, we propose an experiment to address the most pressing one.

*Open Question 1: does spike simultaneity induced by frame-based prostheses affect perception?* Under readily achievable circumstances, a single stimulation pulse can elicit a single action potential, and we can therefore conceive of visual prostheses that control neural activity on a spike-by-spike, perhaps even cell-by-cell basis. Is it important to address millisecond-scale timing between electrodes in order to create high-fidelity, naturalistic perception? Is the application of stimulation in relation to the video frame refresh important? Does the merging of fine time scales created by per-frame binning affect perception?

*Open Question 2: will attention to stimulation pulse timing be necessary to address apparent-motion wobbles that are expected to appear with prostheses supporting higher resolutions?* Recording artifacts in contemporary digital cameras that have rolling shutters, as described above, underscore a potential source of perceptual distortion for devices that use raster-like stimulation patterns.

*Open Question 3: could spike simultaneity be the mechanism of object binding?* Despite substantial evidence that fine-scale spike timing is present in the visual system, the understanding of its role in perception remains largely speculative.

*Open Question 4: if spike synchrony drives object binding, can it therefore be used as a tool in visual prosthesis design?* An artificial vision system provides a model where the role of spike synchrony can be addressed through experimental manipulation, and may be a means to enhance artificial perception. We discuss this idea in more detail in the next section.

### 2.10. Proposed investigation on perceptual impact: object binding and figure-ground separation?

Efforts to answer the question as to whether synchronized or asynchronous stimulation is preferable have been placed primarily on determining low-level factors as reviewed above without considering the impact on higher-level function and utility of visual prostheses. This focus has left open the question of which approach will provide a better real-world experience for recipients of visual prostheses.

While investigation with implanted human subjects would appear to be the most expedient model to determine the relative perceptual merits of synchronous versus asynchronous stimulation, the potential dangers involved, especially for cortical implants (Good et al 2009, Desai et al 2016), suggest that primary animal investigation is warranted. In addition, while answering this question may impact revised versions of current clinical devices, the limited capabilities of contemporary systems including, importantly, low electrode count (Stingl et al 2013, Zrenner 2013, Hornig et al 2017, Bloch et al 2019, Mirochnik and Pezaris 2019, Bloch and da Cruz 2020) suggest that investigation into perceptual issues like object binding with complex images requires the higher number of contacts currently available only at the pre-clinical level (Vurro et al 2014, Killian et al 2016). We therefore propose investigation in non-human primates that will answer these questions by measuring the effects of varying the degree of fine-scale synchronization between activated electrodes in a perceptual recognition task.

As a simplified example, assume the perceptual stimulus to be conveyed is a square (figure 1). The electrodes to be stimulated could be activated all at once, or could be temporally scattered with individual electrodes activated in an interleaved fashion at different points along a variable-length temporal window. As that window length is increased, at what point does the cohesive perception of a square dissolve into visual noise? At what level of asynchrony does object binding fail to occur? And, as a converse corollary, against a visual background of noise from random electrode activation, within what temporal window do phosphenes begin to assemble into a unified object? If this hypothetical perceptual phase change can be exploited, will, therefore, temporal synchronization be a tool to, for example, enhance figure-ground separation in artificial vision akin to confocal imaging (Jung et al 2015)? Will understanding these effects then provide insight into normal function of the brain when we look at precise timing of activity across large ensembles of neurons in each visual area?

## 3. Conclusion

Understanding how spatiotemporal interactions modulate the generation of individual phosphenes and their combination into perceived objects and visual scenes is essential for successful translation of advanced visual prosthesis designs to clinic. In particular, synchronous versus asynchronous electrical stimulation presents a parameter along which various low-level sensory effects have been observed, including super-linear combining effects and sequential inhibition. Some of the undesirable effects of synchronous stimulation may be mitigated through use of local pairs of electrodes to limit electrical fields, allowing it to be used in concert with asynchronous stimulation, potentially creating the opportunity for the use of both strategies in a single device. Discerning the influence of synchronicity on perceptual binding will have strong implications in understanding object perception in normal vision and guide the design of neural prostheses for visual and other sensory modalities.

## Data Availability

The data that support the findings of this study are available upon reasonable request from the authors.

## Glossary

Alpha AMS: A subretinal visual prosthesis (electrode array is implanted within the retinal structures) manufactured by Retina Implant, AG. Carries the CE Mark. No longer available for new implants.
AMD: Age-related macular degeneration. A retinal disease that results in gradual loss of sight, primarily affecting central vision.
Argus II: An epiretinal visual prosthesis (electrode array is applied to the vitral surface of the retina). Manufactured by Second Sight Medical Products. Carries FDA approval and CE Mark. No longer available for new implants.
Artificial Vision: The sensations created by a visual prosthesis (cf) that convey the sense of sight.
Asynchronous: Two or more events occurring at different moments in time. Here, often used to describe the non-instantaneous application of electrical pulses to two or more stimulating electrodes. Such pulses all have some time between them. Contrast with *synchronous*.
FES: Functional electrical stimulation is a method to generate muscle contractions and therefore body movements through externally-applied electrical current. Often it is used to provide restoration of function in paralyzed individuals.
Flicker fusion: The perception that a visual stimulus has constant brightness despite rapid fluttering of actual brightness. At low frequencies, such changes are easily visible; as they increase in rapidity, they become less visible and eventually the stimulus appears to be unchanging.
Form vision: The ability to perceive objects or patterns with meaning, rather than unstructured visual sensations. Having form vision implies being able to, for example, perceive letter identity from a cluster of phosphenes (cf).
CORTIVIS: An experimental visual prosthesis that uses penetrating microelectrodes in the primary visual cortex. Currently in clinical trials.
Cross-talk: The undesirable effects from one channel of action or information that appear as if presented on another. A well-designed multi-channel system has zero cross-talk, so that each channel is entirely independent. See also *field interactions*.
Field Interactions: The interaction of electrical fields here as they have effect when stimulating neurons through multiple electrodes. Non-zero field interactions imply that the principle of superposition (cf) does not apply. See also *cross-talk*.
Ganged Stimulation: The application of electrical current from a single source to many electrodes in parallel. Ganged stimulation is necessarily synchronous across the connected electrodes.
Glaucoma: A disease of the eye that results in degeneration of the optic nerve that can lead to blindness; often a consequence of diabetes.
IRIS II: An epiretinal visual prosthesis (electrode array implanted on the vitreal surface of the retina). Manufactured by Pixium Vision, S.A. Carries the CE Mark.
LGN: Lateral geniculate nucleus of the thalamus. A structure of the early visual pathway (cf) that is the target of the optic nerve, and serves as a relay station for visual signals from the retina to the primary visual cortex (cf).
Microstimulation: The application of currents in the microampere range to neural electrodes that are often physically small.
RNS System: An experimental visual prosthesis that uses surface electrodes applied to primary visual cortex. Currently in clinical trials.
Orion: An experimental visual prosthesis that stimulates neurons in the primary visual cortex. Manufactured by Second Sight Medical Products. Currently in clinical trials.
Phase shift: The relative time difference between two events in an often repetitive sequence. Usually expressed as a fraction of the cyclic period, or in degrees or radians. In the papers discussed here, is expressed instead in milliseconds.
Phosphene: An artificially-generated visual event. Often assumed to be point or star-like in the context of visual prostheses, and considered the pixels of artificial vision. Each electrode contact in a visual prosthesis typically creates one phosphene.
Principle of superposition: A characteristic of linear systems whereby the result of applying stimulus a and b together is the same as the sum of the results of applying each of a and b alone. Mathematically, such systems can be described as obeying the equation f(a+b)=f(a)+f(b).
Retinotopic Map: The arrangement of the visual field as it is represented across a brain area. Called *retinotopic* because such maps originate in the retina.
RGC: Retinal ganglion cells. The neurons that form the final processing stage of the retina. The collected output fibers (axons) of these cells form the optic nerve.
RP: Retinitis pigmentosa. A retinal disease that results in gradual loss of sight, starting with peripheral vision and resulting in tunnel vision before complete blindness.
Scintillation: The perception that a visual stimulus shimmers as if animated by many points of light.
Sequential Stimulation: The application of electrical current to one electrode after another in a set, often with a fixed amount of time between each activation.
Synchronous: Two or more events occurring at the same moment in time. Here, used to describe the instantaneous application of electrical pulses to two or more stimulating electrodes. Such pulses happen all at the same time. Contrast with *asynchronous*.
Visual prosthesis: A device that provides restoration of visual function to blind individuals. The visual prostheses discussed here are all surgically implanted medical devices.
V1: Also known as primary visual cortex. The first stage of visual processing in the cortex. Receives input from the LGN (cf) through the optic radiation.
